# Supplementary material for: Laboratory evaluation of the regeneration time, efficacy and wash-resistance of PermaNet Dual (a deltamethrin-chlorfenapyr net) against susceptible and pyrethroid-resistant strains of Anopheles gambiae sensu lato
Source: PLoS One. 2024 Aug 29;19(8):e0298513. doi: 10.1371/journal.pone.0298513 (PMC11361417; doi:10.1371/journal.pone.0298513)
Supplement: S1 Table — (DOCX) [file pone.0298513.s001.docx]

**S1 Table. Regeneration time cone bioassay results with the susceptible *Anopheles gambiae* sensu stricto Kisumu strain and pyrethroid-resistant *Anopheles gambiae sensu lato* Covè strain.**

|  | | | **Knockdown** | | | | **Delayed mortality** | | | | | | | | |
| --- | --- | --- | --- | --- | --- | --- | --- | --- | --- | --- | --- | --- | --- | --- | --- |
| **Strain** | **Treatment** | **Timepoint** | **N** | **N KD 60 mins** | **% KD 60 mins** | **95% CIs** | **N dead 24 h** | **% dead 24 h** | **95% CIs** | **N dead 48 h** | **% dead 48 h** | **95% CIs** | **N dead 72 h** | **% dead 72 h** | **95% CIs** |
| Kisumu | Untreated net (control) | Unwashed | 79 | 2 | 2.5 | 0.0-5.9 | 1 | 1.3 | 0.0-3.8 | 1 | 1.3 | 0.0-3.8 | 1 | 1.3 | 0.0-3.8 |
|  |  | Day 0 | 73 | 0 | 0.0 | ̶ | 0 | 0.0 | ̶ | 0 | 0.0 | ̶ | 0 | 0.0 | ̶ |
|  |  | Day 1 | 70 | 0 | 0.0 | ̶ | 0 | 0.0 | ̶ | 0 | 0.0 | ̶ | 0 | 0.0 | ̶ |
|  |  | Day 2 | 74 | 0 | 0.0 | ̶ | 0 | 0.0 | ̶ | 0 | 0.0 | ̶ | 0 | 0.0 | ̶ |
|  |  | Day 3 | 77 | 0 | 0.0 | ̶ | 0 | 0.0 | ̶ | 1 | 1.3 | 0.0-3.8 | 1 | 1.3 | 0.0-3.8 |
|  |  | Day 5 | 77 | 0 | 0.0 | ̶ | 1 | 1.3 | 0.0-3.8 | 1 | 1.3 | 0.0-3.8 | 1 | 1.3 | 0.0-3.8 |
|  |  | Day 7 | 79 | 0 | 0.0 | ̶ | 0 | 0.0 | ̶ | 0 | 0.0 | 0.0-0.0 | 0 | 0.0 | ̶ |
|  | PermaNet Dual | Unwashed | 204 | 185 | 90.7 | 86.7-94.7 | 147 | 72.1 | 65.9-78.3 | 156 | 76.5 | 70.7-82.3 | 158 | 77.5 | 71.8-83.2 |
|  |  | Day 0 | 205 | 181 | 88.3 | 83.9-92.7 | 162 | 79.0 | 73.4-84.6 | 168 | 82.0 | 76.7-87.3 | 180 | 87.8 | 83.3-92.3 |
|  |  | Day 1 | 195 | 157 | 80.5 | 74.9-86.1 | 160 | 82.1 | 76.7-87.5 | 169 | 86.7 | 81.9-91.5 | 174 | 89.2 | 84.8-93.6 |
|  |  | Day 2 | 207 | 148 | 71.5 | 65.4-77.6 | 165 | 79.7 | 74.2-85.2 | 178 | 86.0 | 81.3-90.7 | 184 | 88.9 | 84.6-93.2 |
|  |  | Day 3 | 195 | 140 | 71.8 | 65.5-78.1 | 155 | 79.5 | 73.8-85.2 | 159 | 81.5 | 76.0-87.0 | 165 | 84.6 | 79.5-89.7 |
|  |  | Day 5 | 215 | 186 | 86.5 | 81.9-91.1 | 185 | 86.0 | 81.4-90.6 | 198 | 92.1 | 88.5-95.7 | 202 | 94.0 | 90.8-97.2 |
|  |  | Day 7 | 201 | 155 | 77.1 | 71.3-82.9 | 158 | 78.6 | 72.9-84.3 | 162 | 80.6 | 75.1-86.1 | 168 | 83.6 | 78.5-88.7 |
| Covè | Untreated net (control) | Unwashed | 83 | 0 | 0 | ̶ | 1 | 1.2 | 0.0-3.5 | 1 | 1.2 | 0.0-3.5 | 1 | 1.2 | 0.0-3.5 |
|  | PermaNet Dual | Unwashed | 199 | 2 | 1 | 0.0-2.4 | 4 | 2 | 0.1-3.9 | 9 | 4.5 | 1.6-7.4 | 13 | 6.5 | 3.1-9.9 |
