# Supplementary material for: Laboratory evaluation of the regeneration time, efficacy and wash-resistance of PermaNet Dual (a deltamethrin-chlorfenapyr net) against susceptible and pyrethroid-resistant strains of Anopheles gambiae sensu lato
Source: PLoS One. 2024 Aug 29;19(8):e0298513. doi: 10.1371/journal.pone.0298513 (PMC11361417; doi:10.1371/journal.pone.0298513)
Supplement: S2 Table — (DOCX) [file pone.0298513.s002.docx]

**S2 Table. Regeneration time tunnel test results with the pyrethroid-resistant *Anopheles gambiae sensu lato* Covè strain.**

|  |  | **Passage** | | | | **Blood-feeding** | | | | **Delayed mortality** | | | | | | | | | | | |
| --- | --- | --- | --- | --- | --- | --- | --- | --- | --- | --- | --- | --- | --- | --- | --- | --- | --- | --- | --- | --- | --- |
| **Treatment** | **Timepoint** | **N** | **N pass** | **% Pass** | **95% CIs** | **N blf** | **% blf** | **95% CIs** | **% blf inhib** | **N dead imm** | **% dead imm** | **95% CIs** | **N dead 24 h** | **% dead 24 h** | **95% CIs** | **N dead 48 h** | **% dead 48 h** | **95% CIs** | **N dead 72 h** | **% dead 72 h** | **95% CIs** |
| **Untreated net (control)** | **Unwashed** | 95 | 51 | 53.7 | 43.7-63.7 | 64 | 67.4 | 58.0-76.8 | ̶ | 2 | 2.1 | 0.0-5.0 | 2 | 2.1 | 0.0-5.0 | 5 | 5.3 | 0.8-9.8 | 5 | 5.3 | 0.8-9.8 |
|  | **Day 0** | 101 | 71 | 70.3 | 61.4-79.2 | 82 | 81.2 | 73.6-88.8 | ̶ | 2 | 2.0 | 0.0-4.7 | 3 | 3.0 | 0.0-6.3 | 4 | 4.0 | 0.2-7.8 | 4 | 4.0 | 0.2-7.8 |
|  | **Day 1** | 88 | 40 | 45.5 | 35.1-55.9 | 66 | 75 | 66.0-84.0 | ̶ | 2 | 2.3 | 0.0-5.4 | 4 | 4.5 | 0.2-8.8 | 4 | 4.5 | 0.2-8.8 | 4 | 4.5 | 0.2-8.8 |
|  | **Day 2** | 111 | 36 | 32.4 | 23.7-41.1 | 74 | 66.7 | 57.9-75.5 | ̶ | 3 | 2.7 | 0.0-5.7 | 4 | 3.6 | 0.1-7.1 | 4 | 3.6 | 0.1-7.1 | 4 | 3.6 | 0.1-7.1 |
|  | **Day 3** | 102 | 31 | 30.4 | 21.5-39.3 | 82 | 80.4 | 72.7-88.1 | ̶ | 2 | 2.0 | 0.0-4.7 | 3 | 2.9 | 0.0-6.2 | 3 | 2.9 | 0.0-6.2 | 3 | 2.9 | 0.0-6.2 |
|  | **Day 5** | 87 | 51 | 58.6 | 48.2-69.0 | 67 | 77.0 | 68.2-85.8 | ̶ | 2 | 2.3 | 0.0-5.4 | 3 | 3.4 | 0.0-7.2 | 3 | 3.4 | 0.0-7.2 | 3 | 3.4 | 0.0-7.2 |
|  | **Day 7** | 81 | 31 | 38.3 | 27.7-48.9 | 58 | 71.6 | 61.8-81.4 | ̶ | 2 | 2.5 | 0.0-5.9 | 2 | 2.5 | 0.0-5.9 | 2 | 2.5 | 0.0-5.9 | 2 | 2.5 | 0.0-5.9 |
| **PermaNet Dual** | **Unwashed** | 203 | 17 | 8.4 | 4.6-12.2 | 9 | 4.4 | 1.6-7.2 | 93.5 | 190 | 93.6 | 90.2-97.0 | 200 | 98.5 | 96.8-100 | 200 | 98.5 | 96.8-100 | 200 | 98.5 | 96.8-100 |
|  | **Day 0** | 209 | 65 | 31.1 | 24.8-37.4 | 45 | 21.5 | 15.9-27.1 | 73.5 | 165 | 78.9 | 73.4-84.4 | 196 | 93.8 | 90.5-97.1 | 196 | 93.8 | 90.5-97.1 | 198 | 94.7 | 91.7-97.7 |
|  | **Day 1** | 232 | 46 | 19.8 | 14.7-24.9 | 26 | 11.2 | 7.1-15.3 | 85.1 | 223 | 96.1 | 93.6-98.6 | 228 | 98.3 | 96.6-100 | 228 | 98.3 | 96.6-100 | 228 | 98.3 | 96.6-100 |
|  | **Day 2** | 213 | 95 | 44.6 | 37.9-51.3 | 59 | 27.7 | 21.7-33.7 | 58.5 | 182 | 85.4 | 80.7-90.1 | 197 | 92.5 | 89.0-96.0 | 197 | 92.5 | 89.0-96.0 | 197 | 92.5 | 89.0-96.0 |
|  | **Day 3** | 221 | 36 | 16.3 | 11.4-21.2 | 43 | 19.5 | 14.3-24.7 | 75.7 | 148 | 67.0 | 60.8-73.2 | 204 | 92.3 | 88.8-95.8 | 204 | 92.3 | 88.8-95.8 | 204 | 92.3 | 88.8-95.8 |
|  | **Day 5** | 166 | 52 | 31.3 | 24.2-38.4 | 22 | 13.3 | 8.1-18.5 | 82.7 | 152 | 91.6 | 87.4-95.8 | 159 | 95.8 | 92.7-98.9 | 159 | 95.8 | 92.7-98.9 | 159 | 95.8 | 92.7-98.9 |
|  | **Day 7** | 190 | 49 | 25.8 | 19.6-32.0 | 27 | 14.2 | 9.2-19.2 | 80.2 | 157 | 82.6 | 77.2-88.0 | 181 | 95.3 | 92.3-98.3 | 181 | 95.3 | 92.3-98.3 | 181 | 95.3 | 92.3-98.3 |
