# Supplementary material for: Laboratory evaluation of the regeneration time, efficacy and wash-resistance of PermaNet Dual (a deltamethrin-chlorfenapyr net) against susceptible and pyrethroid-resistant strains of Anopheles gambiae sensu lato
Source: PLoS One. 2024 Aug 29;19(8):e0298513. doi: 10.1371/journal.pone.0298513 (PMC11361417; doi:10.1371/journal.pone.0298513)
Supplement: S3 Table — (DOCX) [file pone.0298513.s003.docx]

**S3 Table.** **Wash-resistance cone bioassay results with the susceptible *Anopheles gambiae sensu stricto* Kisumu strain.**

| **Treatment** | **No. of washes** | **N** | **N KD 60 mins** | **% KD 60 mins** | **95% CIs** | **N dead 24 h** | **% dead 24 h** | **95% CIs** | **N dead 48 h** | **% dead 48 h** | **95% CIs** | **N dead 72 h** | **% dead 72 h** | **95% CIs** |
| --- | --- | --- | --- | --- | --- | --- | --- | --- | --- | --- | --- | --- | --- | --- |
| **Untreated net (control)** | **̶** | 403 | 0 | 0.0 | ̶ | 3 | 0.7 | 0.0-1.5 | 5 | 1.2 | 0.1-2.3 | 6 | 1.5 | 0.3-2.7 |
| **PermaNet 2.0** | **0 washes** | 196 | 117 | 59.7 | 52.8-66.6 | 86 | 43.9 | 37.0-50.8 | 92 | 46.9 | 39.9-53.9 | 103 | 52.6 | 45.6-59.6 |
|  | **1 wash** | 199 | 166 | 83.4 | 78.2-88.6 | 131 | 65.8 | 59.2-72.4 | 154 | 77.4 | 71.6-83.2 | 167 | 83.9 | 78.8-89.0 |
|  | **3 washes** | 203 | 176 | 86.7 | 82.0-91.4 | 176 | 86.7 | 82.0-91.4 | 184 | 90.6 | 86.6-94.6 | 187 | 92.1 | 88.4-95.8 |
|  | **5 washes** | 199 | 169 | 84.9 | 79.9-89.9 | 175 | 87.9 | 83.4-92.4 | 178 | 89.4 | 85.1-93.7 | 182 | 91.5 | 87.6-95.4 |
|  | **10 washes** | 194 | 172 | 88.7 | 84.2-93.2 | 160 | 82.5 | 77.2-87.8 | 175 | 90.2 | 86.0-94.4 | 183 | 94.3 | 91.0-97.6 |
|  | **15 washes** | 204 | 178 | 87.3 | 82.7-91.9 | 163 | 79.9 | 74.4-85.4 | 171 | 83.8 | 78.7-88.9 | 182 | 89.2 | 84.9-93.5 |
|  | **20 washes** | 204 | 169 | 82.8 | 77.6-88.0 | 147 | 72.1 | 65.9-78.3 | 159 | 77.9 | 72.2-83.6 | 172 | 84.3 | 79.3-89.3 |
| **Interceptor G2** | **0 washes** | 197 | 73 | 37.1 | 30.4-43.8 | 65 | 33.0 | 26.4-39.6 | 80 | 40.6 | 33.7-47.5 | 87 | 44.2 | 37.3-51.1 |
|  | **1 wash** | 204 | 95 | 46.6 | 39.8-53.4 | 34 | 16.7 | 11.6-21.8 | 39 | 19.1 | 13.7-24.5 | 60 | 29.4 | 23.1-35.7 |
|  | **3 washes** | 201 | 121 | 60.2 | 53.4-67.0 | 68 | 33.8 | 27.3-40.3 | 87 | 43.3 | 36.4-50.2 | 98 | 48.8 | 41.9-55.7 |
|  | **5 washes** | 198 | 131 | 66.2 | 59.6-72.8 | 36 | 18.2 | 12.8-23.6 | 56 | 28.3 | 22.0-34.6 | 70 | 35.4 | 28.7-42.1 |
|  | **10 washes** | 203 | 160 | 78.8 | 73.2-84.4 | 83 | 40.9 | 34.1-47.7 | 89 | 43.8 | 37.0-50.6 | 105 | 51.7 | 44.8-58.6 |
|  | **15 washes** | 200 | 165 | 82.5 | 77.2-87.8 | 106 | 53.0 | 46.1-59.9 | 108 | 54.0 | 47.1-60.9 | 117 | 58.5 | 51.7-65.3 |
|  | **20 washes** | 203 | 167 | 82.3 | 77.0-87.6 | 91 | 44.8 | 38.0-51.6 | 104 | 51.2 | 44.3-58.1 | 123 | 60.6 | 53.9-67.3 |
| **PermaNet Dual** | **0 washes** | 201 | 156 | 77.6 | 71.8-83.4 | 78 | 38.8 | 32.1-45.5 | 85 | 42.3 | 35.5-49.1 | 89 | 44.3 | 37.4-51.2 |
|  | **1 wash** | 196 | 172 | 87.8 | 83.2-92.4 | 135 | 68.9 | 62.4-75.4 | 154 | 78.6 | 72.9-84.3 | 167 | 85.2 | 80.2-90.2 |
|  | **3 washes** | 199 | 161 | 80.9 | 75.4-86.4 | 152 | 76.4 | 70.5-82.3 | 159 | 79.9 | 74.3-85.5 | 161 | 80.9 | 75.4-86.4 |
|  | **5 washes** | 201 | 168 | 83.6 | 78.5-88.7 | 153 | 76.1 | 70.2-82.0 | 162 | 80.6 | 75.1-86.1 | 170 | 84.6 | 79.6-89.6 |
|  | **10 washes** | 203 | 172 | 84.7 | 79.7-89.7 | 142 | 70.0 | 63.7-76.3 | 155 | 76.4 | 70.6-82.2 | 166 | 81.8 | 76.5-87.1 |
|  | **15 washes** | 204 | 169 | 82.8 | 77.6-88.0 | 149 | 73.0 | 66.9-79.1 | 161 | 78.9 | 73.3-84.5 | 175 | 85.8 | 81.0-90.6 |
|  | **20 washes** | 204 | 152 | 74.5 | 68.5-80.5 | 93 | 45.6 | 38.8-52.4 | 110 | 53.9 | 47.1-60.7 | 128 | 62.7 | 56.1-69.3 |
