# Supplementary material for: Laboratory evaluation of the regeneration time, efficacy and wash-resistance of PermaNet Dual (a deltamethrin-chlorfenapyr net) against susceptible and pyrethroid-resistant strains of Anopheles gambiae sensu lato
Source: PLoS One. 2024 Aug 29;19(8):e0298513. doi: 10.1371/journal.pone.0298513 (PMC11361417; doi:10.1371/journal.pone.0298513)
Supplement: S4 Table — (DOCX) [file pone.0298513.s004.docx]

**S4 Table. Wash-resistance tunnel test results with the susceptible *Anopheles gambiae sensu stricto* Kisumu strain and the pyrethroid-resistant *Anopheles gambiae sensu lato* Covè strain.**

|  |  |  | **Passage** | | | | **Blood-feeding** | | | | **Delayed mortality** | | | | | | | | | | | |
| --- | --- | --- | --- | --- | --- | --- | --- | --- | --- | --- | --- | --- | --- | --- | --- | --- | --- | --- | --- | --- | --- | --- |
| **Strain** | **Treatment** | **No. of washes** | **N** | **N pass** | **% pass** | **95% CIs** | **N blf** | **% blf** | **95% CIs** | **% blf inhib** | **N dead imm** | **% dead imm** | **95% CIs** | **N dead 24 h** | **% dead 24 h** | **95% CIs** | **N dead 48 h** | **% dead 48 h** | **95% CIs** | **N dead 72 h** | **% dead 72 h** | **95% CIs** |
| **Kisumu** | **Untreated net (control)** | **̶** | 279 | 173 | 62.0 | 56.3-67.7 | 219 | 78.5 | 73.7-83.3 | ̶ | 6 | 2.2 | 0.5-3.9 | 8 | 2.9 | 0.9-4.9 | 12 | 4.3 | 1.9-6.7 | 12 | 4.3 | 1.9-6.7 |
|  | **PermaNet 2.0** | **0 washes** | 226 | 80 | 35.4 | 29.2-41.6 | 0 | 0.0 | ̶ | 100 | 219 | 96.9 | 94.6-99.2 | 224 | 99.1 | 97.9-100 | 224 | 99.1 | 97.9-100 | 225 | 99.6 | 98.8-100 |
|  |  | **10 washes** | 227 | 61 | 26.9 | 21.1-32.7 | 4 | 1.8 | 0.1-3.5 | 97.7 | 227 | 100 | ̶ | 227 | 100 | ̶ | 227 | 100 | ̶ | 227 | 100 | ̶ |
|  |  | **20 washes** | 204 | 51 | 25.0 | 19.1-30.9 | 2 | 1.0 | 0.0-2.4 | 98.7 | 203 | 99.5 | 98.5-100 | 203 | 99.5 | 98.5-100 | 203 | 99.5 | 98.5-100 | 203 | 99.5 | 98.5-100 |
|  | **Interceptor G2** | **0 washes** | 201 | 95 | 47.3 | 40.4-54.2 | 19 | 9.5 | 5.4-13.6 | 87.9 | 196 | 97.5 | 95.3-99.7 | 199 | 99.0 | 97.6-100 | 200 | 99.5 | 98.5-100 | 200 | 99.5 | 98.5-100 |
|  |  | **10 washes** | 230 | 80 | 34.8 | 28.6-41.0 | 2 | 0.9 | 0.0-2.1 | 98.9 | 227 | 98.7 | 97.2-100 | 228 | 99.1 | 97.9-100 | 229 | 99.6 | 98.8-100 | 229 | 99.6 | 98.8-100 |
|  |  | **20 washes** | 218 | 98 | 45.0 | 38.4-51.6 | 3 | 1.4 | 0.0-3.0 | 98.2 | 216 | 99.1 | 97.8-100 | 216 | 99.1 | 97.8-100 | 216 | 99.1 | 97.8-100 | 216 | 99.1 | 97.8-100 |
|  | **PermaNet Dual** | **0 washes** | 193 | 125 | 64.8 | 58.1-71.5 | 10 | 5.2 | 2.1-8.3 | 93.4 | 193 | 100 | ̶ | 193 | 100 | ̶ | 193 | 100 | ̶ | 193 | 100 | ̶ |
|  |  | **10 washes** | 214 | 93 | 43.5 | 36.9-50.1 | 23 | 10.7 | 6.6-14.8 | 86.4 | 214 | 100 | ̶ | 214 | 100 | ̶ | 214 | 100 | ̶ | 214 | 100 | ̶ |
|  |  | **20 washes** | 220 | 76 | 34.5 | 28.2-40.8 | 3 | 1.4 | 0.0-3.0 | 98.2 | 209 | 95.0 | 92.1-97.9 | 215 | 97.7 | 95.7-99.7 | 216 | 98.2 | 96.4-100 | 216 | 98.2 | 96.4-100 |
| **Covè** | **Untreated net (control)** | **̶** | 286 | 141 | 49.3 | 43.5-55.1 | 218 | 76.2 | 71.3-81.1 | ̶ | 8 | 2.8 | 0.9-4.7 | 12 | 4.2 | 1.9-6.5 | 12 | 4.2 | 1.9-6.5 | 12 | 4.2 | 1.9-6.5 |
|  | **PermaNet 2.0** | **0 washes** | 186 | 66 | 35.5 | 28.6-42.4 | 12 | 6.5 | 3.0-10.0 | 91.5 | 137 | 73.7 | 67.4-80.0 | 153 | 82.3 | 76.8-87.8 | 156 | 83.9 | 78.6-89.2 | 160 | 86.0 | 81.0-91.0 |
|  |  | **10 washes** | 227 | 75 | 33.0 | 26.9-39.1 | 28 | 12.3 | 8.0-16.6 | 83.9 | 89 | 39.2 | 32.8-45.6 | 121 | 53.3 | 46.8-59.8 | 132 | 58.1 | 51.7-64.5 | 146 | 64.3 | 58.1-70.5 |
|  |  | **20 washes** | 211 | 129 | 61.1 | 54.5-67.7 | 51 | 24.2 | 18.4-30.0 | 68.2 | 67 | 31.8 | 25.5-38.1 | 92 | 43.6 | 36.9-50.3 | 98 | 46.4 | 39.7-53.1 | 103 | 48.8 | 42.1-55.5 |
|  | **Interceptor G2** | **0 washes** | 180 | 39 | 21.7 | 15.7-27.7 | 9 | 5.0 | 1.8-8.2 | 93.4 | 175 | 97.2 | 94.8-99.6 | 179 | 99.4 | 98.3-100 | 179 | 99.4 | 98.3-100 | 179 | 99.4 | 98.3-100 |
|  |  | **10 washes** | 190 | 62 | 32.6 | 25.9-39.3 | 26 | 13.7 | 8.8-18.6 | 82.0 | 159 | 83.7 | 78.4-89.0 | 175 | 92.1 | 88.3-95.9 | 175 | 92.1 | 88.3-95.9 | 176 | 92.6 | 88.9-96.3 |
|  |  | **20 washes** | 233 | 64 | 27.5 | 21.8-33.2 | 38 | 16.3 | 11.6-21.0 | 78.6 | 162 | 69.5 | 63.6-75.4 | 200 | 85.8 | 81.3-90.3 | 200 | 85.8 | 81.3-90.3 | 204 | 87.6 | 83.4-91.8 |
|  | **PermaNet Dual** | **0 washes** | 207 | 46 | 22.2 | 16.5-27.9 | 11 | 5.3 | 2.2-8.4 | 93.0 | 200 | 96.6 | 94.1-99.1 | 203 | 98.1 | 96.2-100 | 203 | 98.1 | 96.2-100 | 203 | 98.1 | 96.2-100 |
|  |  | **10 washes** | 235 | 49 | 20.9 | 15.7-26.1 | 32 | 13.6 | 9.2-18.0 | 82.2 | 218 | 92.8 | 89.5-96.1 | 228 | 97.0 | 94.8-99.2 | 228 | 97.0 | 94.8-99.2 | 229 | 97.4 | 95.4-99.4 |
|  |  | **20 washes** | 212 | 55 | 25.9 | 20.0-31.8 | 43 | 20.3 | 14.9-25.7 | 73.4 | 159 | 75.0 | 69.2-80.8 | 179 | 84.4 | 79.5-89.3 | 188 | 88.7 | 84.4-93.0 | 194 | 91.5 | 87.7-95.3 |
