# Supplementary material for: Laboratory evaluation of the regeneration time, efficacy and wash-resistance of PermaNet Dual (a deltamethrin-chlorfenapyr net) against susceptible and pyrethroid-resistant strains of Anopheles gambiae sensu lato
Source: PLoS One. 2024 Aug 29;19(8):e0298513. doi: 10.1371/journal.pone.0298513 (PMC11361417; doi:10.1371/journal.pone.0298513)
Supplement: S5 Table — (DOCX) [file pone.0298513.s005.docx]

**S5 Table. Wash-retention of active ingredient content in PermaNet® Dual, PermaNet® 2.0 and Interceptor® G2 net pieces used in regeneration time and wash-resistance studies.**

| **Study type** | **Net type** | **Active ingredient (AI)** | **No. of washes** | **AI content (g/kg)*** | **% Relative standard deviation (RSD)** | **% AI retention (of wash 0)** | **% Wash-resistance index (at each wash)** |
| --- | --- | --- | --- | --- | --- | --- | --- |
| Regeneration time | PermaNet Dual | Deltamethrin | 0 | 2.14 | ̶ | ̶ | ̶ |
|  |  |  | 3 | 1.82 | ̶ | 85.1 | 94.7 |
|  |  | Chlorfenapyr | 0 | 4.33 | ̶ | ̶ | ̶ |
|  |  |  | 3 | 3.03 | ̶ | 70.0 | 88.8 |
| Wash-resistance | PermaNet 2.0 | Deltamethrin | 0 | 1.29 | 2.7 | ̶ | ̶ |
|  |  |  | 1 | 1.20 | 2.1 | 92.3 | 92.3 |
|  |  |  | 3 | 0.99 | 4.7 | 76.5 | 91.4 |
|  |  |  | 5 | 0.79 | 4.7 | 61.2 | 90.6 |
|  |  |  | 10 | 0.46 | 1.3 | 35.7 | 90.2 |
|  |  |  | 15 | 0.29 | 10.9 | 22.4 | 90.5 |
|  |  |  | 20 | 0.15 | 10.9 | 11.8 | 89.9 |
|  | Interceptor G2 | Alpha-cypermethrin | 0 | 2.51 | 20.7 | ̶ | ̶ |
|  |  |  | 1 | 2.77 | 16.6 | 110.6 | 110.6 |
|  |  |  | 3 | 2.74 | 21.2 | 109.1 | 103.0 |
|  |  |  | 5 | 2.13 | 24.6 | 85.1 | 96.8 |
|  |  |  | 10 | 2.06 | 17.8 | 82.3 | 98.1 |
|  |  |  | 15 | 1.64 | 6.6 | 65.4 | 97.2 |
|  |  |  | 20 | 1.65 | 25.9 | 65.6 | 97.9 |
|  |  | Chlorfenapyr | 0 | 5.64 | 23.1 | ̶ | ̶ |
|  |  |  | 1 | 6.13 | 14.5 | 108.7 | 108.7 |
|  |  |  | 3 | 5.27 | 19.4 | 93.5 | 97.8 |
|  |  |  | 5 | 3.76 | 29.7 | 66.7 | 92.2 |
|  |  |  | 10 | 2.92 | 20.2 | 51.7 | 93.6 |
|  |  |  | 15 | 1.93 | 9.2 | 34.2 | 93.1 |
|  |  |  | 20 | 1.67 | 43.8 | 29.7 | 94.1 |
|  | PermaNet Dual | Deltamethrin | 0 | 2.15 | 8.6 | ̶ | ̶ |
|  |  |  | 1 | 1.97 | 5.9 | 91.7 | 91.7 |
|  |  |  | 3 | 1.79 | 5.0 | 83.2 | 94.0 |
|  |  |  | 5 | 1.59 | 11.1 | 74.1 | 94.2 |
|  |  |  | 10 | 1.14 | 4.9 | 53.1 | 93.9 |
|  |  |  | 15 | 0.88 | 19.7 | 40.8 | 94.2 |
|  |  |  | 20 | 0.52 | 16.2 | 24.2 | 93.2 |
|  |  | Chlorfenapyr | 0 | 4.30 | 17.1 | ̶ | ̶ |
|  |  |  | 1 | 4.13 | 6.3 | 96.1 | 96.1 |
|  |  |  | 3 | 3.18 | 10.4 | 73.9 | 90.4 |
|  |  |  | 5 | 2.45 | 14.0 | 56.9 | 89.3 |
|  |  |  | 10 | 0.96 | 10.2 | 22.4 | 86.1 |
|  |  |  | 15 | 0.58 | 57.7 | 13.6 | 87.5 |
|  |  |  | 20 | 0.23 | 20.9 | 5.4 | 86.4 |
